# Incidence of tuberculosis in patients with immune-mediated diseases undergone biological therapy: A 10-year observational study in a high-burden region of northeastern Brazil

**DOI:** 10.1371/journal.pone.0353691

**Published:** 2026-07-27

**Authors:** Rosinete Maria dos Santos Guimarães, Claudia Diniz Lopes Marques, Ulisses Ramos Montarroyos, Carolina Santos Guimarães, Jaqueline Alves da Silva, Linaldo Francisco Silva Filho, Vanessa Medeiros, Irassandra Rooze Pereira Uchôa Cavalcanti de Aquino, Rafael Dhalia, Edmundo Pessoa Lopes, Carlos Alexandre Antunes de Brito

**Affiliations:** 1 Center for Medical Sciences, Postgraduate Program in Tropical Medicine, Federal University of Pernambuco, Recife, Pernambuco, Brazil; 2 Center for Medical Sciences, Postgraduate Program in Translational Health, Federal University of Pernambuco, Recife, Pernambuco, Brazil; 3 Institute of Biological Sciences, Postgraduate Program in Health Sciences, University of Pernambuco, Recife, Pernambuco, Brazil; 4 School of Medicine, Maurício de Nassau University, Recife, Pernambuco, Brazil; 5 Coordination of the State Tuberculosis Control Program, State Health Department of Pernambuco, Recife, Pernambuco, Brazil; 6 Executive Department for Health Care, State Health Department of Pernambuco, General Directorate of Pharmaceutical Assistance, Recife, Pernambuco, Brazil; 7 Department of Dermatology, Federal University of Pernambuco, Recife, Pernambuco, Brazil; 8 Autoimune Research Institute, Recife, Pernambuco, Brazil; 9 Aggeu Magalhães Institute – Fiocruz, Recife, Pernambuco, Brazil; 10 Federal University of Pernambuco, Hospital das Clínicas/HU Brasil, Recife, Pernambuco, Brazil; Nippon Medical School, JAPAN

## Abstract

Biological therapies are critical in the management of Immune-Mediated Diseases (IMDs), yet their use is linked to adverse effects, including an elevated risk of Tuberculosis (TB), a significant health issue in endemic regions like Brazil. This study aimed to assess the incidence of TB among patients with IMDs in rheumatology, gastroenterology, and dermatology who received biological therapy in Pernambuco between 2013 and 2023. A total of 8,623 patients were analyzed, with 90.7% diagnosed with rheumatologic diseases, 5.6% with gastroenterological diseases, and 3.7% with psoriasis. In the descriptive analysis, frequency distributions and medians with interquartile ranges were reported, and Relative Risk (RR) was calculated to compare tuberculosis occurrence across IMD groups. The overall TB incidence was 2.1%, highest in gastroenterological patients (4.7%) compared to rheumatologic (2.0%) and psoriasis patients (1.3%) with a risk ratio (RR) of 2.4 for the group of gastroenterological diseases (p < 0.001). No significant differences in TB frequency were detected before (1.16%) or during (0.97%) the use of biological therapy, nor among drug classes, except for a slightly higher incidence with infliximab (2.6%). The absence of differences between the other anti-Tumor Necrosis Factor (anti-TNF) drugs and other cytokine inhibitors suggests that TB incidence might be influenced by environmental exposure in endemic regions. Pulmonary TB was the predominant form (80.8%), and pleural TB emerged as the most frequent extrapulmonary manifestation. The results underscore the need for extensive regional studies to elucidate TB risks in IMD patients and refine treatment guidelines for immunobiological therapies.

## Introduction

Tuberculosis (TB), a preventable and treatable infectious disease caused by *Mycobacterium tuberculosis*, continues to represent a significant global public health challenge, affecting approximately one-quarter of the world’s population. Brazil is among the 30 countries with the highest number of TB cases [1], reporting 80,012 new cases annually and an incidence rate of 38 per 100,000 inhabitants². Within Brazil, the state of Pernambuco ranks fifth in incidence, with 51.5 cases per 100,000 inhabitants, while its capital, Recife, records 99 cases per 100,000 inhabitants, the third highest rate in the country [2].

The risk of developing TB increases in populations with immunosuppression, including individuals undergoing immunosuppressive therapies, particularly biological therapies [3,4]. Biological therapies are an essential treatment option for immune-mediated diseases (IMDs) in rheumatology, gastroenterology, and dermatology, providing substantial clinical benefits. However, their use is associated with adverse events, including an elevated risk of TB, particularly in regions where TB is endemic [4–7].

The frequency of TB varies by drug class, with higher rates reported in infliximab users compared to etanercept within the anti-Tumor Necrosis Factor (anti-TNF) class [8–10], and lower rates observed with non-anti-TNF cytokine inhibitors. However, these observations are inconsistent, influenced by differences in study design, the rigor of TB screening protocols, and the regional TB incidence where the studies were conducted [9,11–16].

There is limited knowledge about the regional differences in TB clinical presentation, as well as the severity and outcomes of TB in specific populations. Few studies have addressed these elements comprehensively [6,7,10,12,14,17,18]. This study aimed to evaluate the incidence, clinical-epidemiological profile, and outcomes of TB among patients with IMDs in rheumatology, gastroenterology, and dermatology who have received biological therapies in Pernambuco, a Northeastern state of Brazil with high TB incidence rates.

## Materials and methods

### Study design and setting

This study utilized a descriptive, cross-sectional, and retrospective design to analyze a database of patients with immune-mediated diseases (IMDs) who were eligible for biologic therapy and registered to receive medication free of charge through the public drug dispensing system in the state of Pernambuco, located in Northeast Brazil. The analysis spanned the period from January 2, 2013, to January 19, 2023. All patients diagnosed with an IMD and receiving biologic therapy during this period were included.

### Data sources and linkage

These data were cross-referenced with the Brazilian Notifiable Diseases Information System (BNDIS) database for tuberculosis, where all TB cases are mandatorily reported to ensure treatment. The date of TB notification was used to establish the temporal relationship between TB diagnosis and the initiation of biologic therapy in IMD patients. TB cases diagnosed prior to the initiation of biologic therapy were classified as ‘before treatment,’ while those diagnosed after the initiation of biologic therapy or during the period of biologic drug use were classified as ‘during treatment.’ Only incident TB cases in patients with a previously established IMD diagnosis (registered in the official system) were included in the analysis during the study period.

### Data access and healthcare system

Authors had access to information that could identify individual participants, between 10/09/2024 and 05/02/2025, following IDB approval, after the completion of data collection. With a population of approximately nine million, roughly 85% of residents in Pernambuco depend on the public health system for medical care, while the remaining 15% access private healthcare services. This study included only patients with IMDs who were registered in the public healthcare system’s biologic drug dispensing database, which covers approximately 85% of the state’s population.

### Study population and diagnostic criteria

The study population included individuals notified with TB who were eligible for biological therapy across three specialist fields: rheumatology (conditions such as rheumatoid arthritis, ankylosing spondylitis, psoriatic arthritis, and other unspecified spondyloarthropathies), gastroenterology (including ulcerative colitis and Crohn’s disease) and dermatology (psoriasis). Diseases are diagnosed according to the International Classification of Diseases (ICD-10, version: 2019) codes. The healthcare system mandates the submission of medical reports, complementary examinations, and disease activity indices as part of the diagnostic process. These criteria are outlined in clinical protocols and therapeutic guidelines, which are based on scientific evidence, as well as national and international medical recommendations. These guidelines are developed with input from specialists and a national governmental committee, ensuring the reliability of disease diagnoses. The treatments recommended for each ICD-10 condition are aligned with these same protocols. All documents, protocols, and requirements are publicly accessible through the national healthcare platform [19].

### Biologic therapies and exposure assessment

Clinical and epidemiological parameters were analyzed to assess TB incidence within IMD subgroups, as well as across different classes of biologic therapies and individual biologic agents. The biologics investigated included Anti-TNF agents: infliximab, adalimumab, certolizumab, golimumab, and etanercept; cytokine inhibitors: secukinumab (anti-il-17), ustekinumab (anti-il-12/23), and risankizumab (anti-il-23); anti-cd20 monoclonal antibody: rituximab; selective co-stimulation modulator: abatacept; anti-integrin agent: vedolizumab. These biologics were identified from public pharmacy records available in Pernambuco during the study period. The duration of biologic therapy is systematically recorded in the public pharmacy dispensing system at the time of the initial dispensation and is monitored monthly through renewals, which require medical reports confirming treatment continuation. Discontinuation of treatment is also documented via a formal medical report, enabling an accurate assessment of treatment duration.

### Latent tuberculosis screening and management

In Pernambuco, screening and treatment for latent tuberculosis infection (LTBI) adhere to international WHO guidelines, which are aligned with the protocols of the Brazilian Ministry of Health. These guidelines are implemented across both the public healthcare system and the private supplementary health sector. Before initiating any biologic therapy, screening for LTBI is mandatory and includes a tuberculin skin test (TST) or an interferon-gamma release assay (IGRA), along with a chest radiograph. Biologic therapies are authorized only after the completion of this screening, and if LTBI is detected, treatment must be initiated. Importantly, LTBI treatment is provided exclusively through government programs and is accessible to patients in both public and private healthcare services.

### Statistical analysis

The descriptive analysis of sociodemographic and clinical characteristics included frequency distributions for categorical variables and medians with interquartile ranges for continuous variables. Tuberculosis incidence among IMD patients was estimated with a 95% confidence interval. Relative Risk (RR) was calculated to assess the association between tuberculosis and IMD groups. RR was estimated using a logistic regression model, where the measure of association obtained, the odds ratio (OR), was interpreted as an estimate of RR. Since tuberculosis is a low-frequency/incidence condition in IMD patients, the odds ratio estimated by the model closely approximates the relative risk [20]. Rheumatologic diseases were selected as the reference group for calculating relative risk. This decision was based on a statistical rationale, choosing the category with the largest sample size to enhance the precision of the estimates. Pearson’s chi-square test was utilized for association analyses, and the Kruskal-Wallis test was applied for median comparisons. A significance level of 5% (p < 0.05) was established for the study.

### Residual immunosuppression risk window

To evaluate the potential residual risk of immunosuppression associated with biological therapies following drug discontinuation, TB cases were classified as linked to residual drug-mediated immunosuppression if they occurred within a timeframe corresponding to four to five half-lives of the specific biologic agent. This window was aligned with established guidelines for safety and risk evaluation during live-virus vaccination post-biologic therapy cessation [21–23]. The immunosuppressive risk windows for residual drug activity were categorized as follows: three months for anti-TNF agents (infliximab, adalimumab, certolizumab, golimumab), non-anti-TNF cytokine inhibitors (risankizumab, secukinumab, tocilizumab, ustekinumab), the selective T-cell co-stimulation modulator (abatacept), and anti-integrin agent (vedolizumab); four weeks for etanercept; six months for rituximab [21–23]. By adhering to these defined risk windows, safety assessments of immunosuppression-related TB incidence were standardized to ensure scientific rigor and clinical relevance.

### Ethical issues

This study was approved by the Research Ethics Committee of the Federal University of Pernambuco under protocol number CAAE 80140524.2.0000.5208, in compliance with the guidelines established by the National Health Council for research involving human participants.

## Results

### Study population and tuberculosis frequency

A total of 8,623 patients diagnosed with IMD who had undergone biological therapy were included in the analysis, with their data sourced from the drug distribution system spanned the period from 2013 to 2023. Among this cohort, 182 cases of TB were identified, corresponding to a frequency of 21.0 cases per 1,000 patients with IMIDs (2.1%). The distribution of TB cases was stratified as follows: 155 cases (85.1%) occurred in patients with Immune-Mediated Rheumatologic Diseases (IMRD), 23 cases (12.6%) in patients with Immune-Mediated Gastroenterologic Diseases (IMGD) diseases, and 4 cases (2.3%) in patients with Immune-Mediated Dermatologic Diseases (IMDD) (Table 1).

**Table 1:** Sociodemographic and clinical characteristics of 182 patients with Tuberculosis (TB) in immune-mediated diseases (IMDs) with a history of biological therapy use, in Pernambuco, between 2013 and 2023.

Features^a^	With tuberculosis (N = 182)	TB in immune-mediated diseases			Without Tuberculosis (N = 8,441)	P Value^a^	P Value^b^
		Rheumatological Diseases (N = 155)	Gastrointestinal Diseases (N = 23)	Dermatological Diseases (N = 4)			
**Age in January 2023 (in years)**							
Median (P_25_ – P_75_)	49 (39–57)	49 (40–55)	31 (19–53)	35 (20–53)	53 (42–62)	**<0.001**	**<0,001**
**Age range**							
Up to 39 y/o	50 (27.5%)	35 (22.6%)	13 (56.5%)	2 (50.0%)	1,759 (20.8%)	**0.030**	**<0,001**
40–49 y/o	56 (30.8%)	51 (32.9%)	4 (17.4%)	1 (25.0%)	1,747 (20.7%)		
50–59 y/o	47 (25.8%)	44 (28.4%)	3 (13.0%)	0 (0%)	2,130 (25.2%)		
60 years y/o	29 (15.9%)	25 (19.3%)	3 (10.7%)	1 (25.0%)	2,805 (33.2%)		
**Gender**							
Female	106 (58.2%)	93 (60.0%)	13 (56.5%)	0 (0%)	5,868 (69.5%)	0.063	0.001
Male	76 (41.8%)	62 (40.0%)	10 (43.5%)	4 (100%)	2,573 (30.5%)		
**Origin**							
Recife	53 (29.1%)	44 (28.4%)	8 (34.8%)	1 (25.0%)	2,142 (25.4%)	0.735	**0.002**
Metropolitan Regian	61 (29.1%)	50 (32.3%)	9 (39.1%)	2 (50.0%)	2,074 (24.6%)		
State interior	68 (37.4%)	61 (39.3%)	6 (26.1%)	1 (25.0%)	4,225 (50.0%)		
**Treatment duration (years)**							
Median (P_25_ – P_75_)^c^	1.3 (0.4–2.3)	1.3 (0.4–2.3)	1.4 (0.8–1.9)	0.08	3.5 (1.7–6.2)	0.193	**<0,001**

N: Number of patients. y/o: years old; The p-value was determined using Pearson’s chi-square test for association analyses and the Kruskal-Wallis test for median comparisons. The significance level adopted in the study was 5% (p < 0.05).

^a^Difference between IMD groups (rheumatological, gastrointestinal, dermatological).

^b^Difference between tuberculosis groups (with and without tuberculosis).

^c^Duration of biological therapy treatment until TB diagnosis, referring to the 83 TB cases among patients with IMD (69 cases in IMRD, 13 cases in IMGD, and one case in IMDD). Duration of biological therapy treatment in cases without tuberculosis.

The median age of the study population was 49 years, with statistically significant differences observed between the IMD subgroups (p < 0.001). Patients with IMRD demonstrated the highest median age, while those with IMGD were significantly younger, with 56.6% of the IMGD cohort aged under 39 years (p = 0.030). Regarding gender distribution, 58.2% of the patients were female.

When comparing demographic data between patients with TB and those without TB, the median age was observed to be 49 years in the TB group and 53 years in the non-TB group, indicating a statistically significant difference (p < 0.001). In terms of gender distribution, males accounted for 42.8% (76/182) of TB cases, compared to 30.5% (2,573/8,441) in the non-TB group, also demonstrating statistical significance (p < 0.001).

Regarding the time interval from the initiation of therapy to TB diagnosis, the median duration was 1.3 years (range: 0.4–2.3 years) among the 83 confirmed cases. Of these, 36 cases (43.4%) occurred within the first year, 26 cases (31.3%) were diagnosed between one and two years, and 21 cases (25.3%) were identified beyond two years. No significant differences were found between the IMD subgroups (Table 1).

When comparing the frequency of TB across the IMD subgroups, patients with IMGD exhibited a significantly higher incidence of TB compared to patients with IMRD and IMDD (p < 0.001). Among the 484 patients in the IMGD subgroup who received biological therapy, 23 (4.7%) presented TB. In comparison, 155 TB cases (2%) were identified among 7,822 patients with IMRD, and 4 TB cases (1.3%) among 317 patients with IMD (Table 2).

**Table 2:** Incidence and relative risk of Tuberculosis (TB) by group of immune-mediated diseases (IMDs), in Pernambuco, between 2013 and 2023.

Variables	Number of patients	Incidence^a^ (CI 95%)	Relative Risks^b^ (%)	P Value^c^
**Rheumatological Diseases**	**7.822**	**155 (2.0%)**	**Reference**	**–**
Rheumatoid arthritis	4.789	78 (1.6%)	Reference	–
Psoriatic arthritis	852	7 (0.8%)	0.50 (0.23–1.09)	0.064
Axial Spondyloarthritis	2.181	70 (3.2%)	1.85 (1.37–2.51)	<0.001
**Gastrointestinal Diseases**	**484**	**23 (4.7%)**	**2.4 (1.56–4.69)**	**<0.001**
Crohn’s disease	388	21 (5.4%)	Reference	–
Ulcerative enterocolitis	96	2 (2.1%)	0.38 (0.09–1.61)	0.169
**Dermatological Diseases** **(Psoriasis vulgaris)**	**317**	**4 (1.3%)**	**0.64 (0.24–1.71)**	**0.379**

^a^Incidences by IMD groups (rheumatological, gastrointestinal, dermatological) and by diseases subtype.

^b^The Confidence Interval (CI) with a 95% level of reliability was determined. The Relative Risks found in rheumatoid arthritis (for rheumatological diseases) and Crohn’s Disease (for gastrointestinal diseases) were used as a reference.

^c^The P Value was determined using Pearson’s chi-square test for association analyses and the Kruskal-Wallis test for median comparisons. The significance level adopted in the study was 5% (p < 0.05).

In the age group analysis, we observed a younger population among patients with Axial Spondyloarthritis (AS), with a median age of 45 years, compared to median ages of 56 and 57 years for patients with rheumatoid arthritis and psoriatic arthritis, respectively (p < 0.001). Among patients with AS, the group younger than 40 years was the most prevalent, accounting for 33.8% of the sample. In contrast, among patients with rheumatoid arthritis and psoriatic arthritis, the most prevalent group was those aged 59 years or older, representing 43.4% and 39.6% of the samples, respectively (S1 Table).

### Timing of tuberculosis occurrence

An analysis of the timing of TB onset revealed that 99 cases (1.1%) occurred prior to the initiation of biological therapy, while 83 cases (0.9%) developed during the use of biological therapy. The difference in TB occurrence between the pre-therapy and on-therapy periods was not statistically significant (Table 3).

**Table 3:** Incidence of Tuberculosis (TB) by period of diagnosis of the disease in relation to the treatment of Immune-Mediated Diseases (IMDs), in Pernambuco, between 2013 and 2023.

Groups	Before Treatment^a^ N/Total (% - CI 95%)	During Treatment^b^ N/Ttotal (% - CI 95%)	P Value^c^
**General**	99/8.540 (1.16–0.95 a 1.41)	83/8.524 (0.97–0.78 a 1.21)	0.227
Rheumatological Diseases	86/7.753 (1.11–0.90 a 1.37)	69/7.736 (0.89–0.71 a 1.13)	0.169
Gastrointestinal Diseases	10/471 (2.12–1.14 a 3.91)	**13/474 (2.74–1.60 a 4.67)**	0.536
Dermatological Diseases	3/316 (0.95–0.31 a 2.91)	1/314 (0.32–0.04 a 2.24)	0.320

^a^Incidences by IMD groups (rheumatological, gastrointestinal, dermatological) before treatment.

^b^Incidences by IMD groups (rheumatological, gastrointestinal, dermatological) during treatment.

^c^The p-value was determined using Pearson’s chi-square test for association analyses and the Kruskal-Wallis test for median comparisons. The significance level adopted in the study was 5% (p < 0.05).

*Pre-treatment incidence comparison: Rheumatological vs. Gastrointestinal disease: **p = 0.047**.

**Pre-treatment incidence comparison: Rheumatological vs. Dermatological disease: p = 0.790.

***Pre-treatment incidence comparison: Gastrointestinal vs. Dermatological disease: p = 0.205.

****During treatment incidence comparison: Rheumatological vs. Gastrointestinal disease: p < 0.001.

*****During treatment incidence comparison: Rheumatological vs. Dermatological disease: p = 0.283.

******During treatment incidence comparison: Gastrointestinal vs. Dermatological disease: **p = 0.012.**

In the analysis by therapeutic class groups, even among patients receiving TNFi, the risk of TB did not differ before versus during biologic therapy (Before treatment: 65/6,862 [0.95; 95% CI 0.74–1.21] vs During treatment: 74/6,871 [1.08; 95% CI 0.85–1.35], p = 0.447) (S2 Table).

Among patients on biological therapy, the IMGD subgroup demonstrated the highest incidence of TB, with 13 cases (2.7%) identified. This was markedly higher than the 69 cases (0.9%) observed in the IMRD subgroup, and the single case (0.3%) recorded in the IMD subgroup.

### Tuberculosis frequency by therapeutic classes

Of the 83 patients who developed TB during biological therapy, the majority, 74 (89.2%), were receiving anti-TNF agents, followed by 5 patients (6%) using cytokine inhibitors and 4 patients (4.8%) on anti-CD20 therapy. The frequency of TB incidence by therapeutic class was as follows: anti-TNF agents 1.07% (74/6,936), non-anti-TNF cytokine inhibitors 0.57% (5/879), and anti-CD20 agents 1.07% (4/373).

Within the anti-TNF class, the highest TB incidence at 2.6% (20/789) was associated with infliximab, followed by certolizumab at 1.0% (6/638), adalimumab at 0.9% (26/2,876), golimumab at 0.9% (9/1,012), and etanercept at 0.8% (13/1,621). The incidence of TB among infliximab users was significantly higher compared to all other anti-TNF agents (p < 0.05) and non-anti-TNF cytokine inhibitors, except for rituximab (p = 0.112).

Statistical analysis revealed no significant differences in TB incidence among the remaining anti-TNF agents apart from infliximab. Additionally, comparison of TB incidence across therapeutic drug classes (anti-TNF, cytokine inhibitors, and anti-CD20 agents) did not yield differences (p = 0.138). Similarly, the time interval from the initiation of therapy to TB diagnosis did not differ significantly when assessed across the different TNF inhibitors (Table 4).

**Table 4:** Cases of Tuberculosis (TB) occurring during treatment with biological therapy by type of drug used in patients with immune-mediated diseases (IMDs), in Pernambuco, between 2013 and 2023.

Medications used in the diagnosis of Tuberculosis	Tuberculosis Cases (N = 83)	Total number of patients using therapy	Duration treatment until TB diagnosis (in years) Median (P_25_ – P_75_)	Cases/type of therapy (%)	P Value^a^ (between subclasses)
**TNFi**	**74 (89.2%)**	**6.936**	**1.3 (0.4–2.5)**	**1.07%**	**0.006**
Adalimumab	26 (35.1%)	2.876	1.4 (0.7–2.8)	0.9%	
Certolizumab	6 (8.1%)	638	1.0 (0.4–2.9)	1.0%	
Etanercept	13 (17.6%)	1.621	1.2 (0.2–1.7)	0.8%	
Golimumab	9 (12.2%)	1.012	1.0 (0.3–1.3)	0.9%	
Infliximab	20 (27.0%)	789	1.3 (0.7–3.0)	2.6%	
**Non- TNFi cytokine inhibitor**	**5 (6.0%)**	**879**	**0.4 (0.3–1.3)**	**0.57%**	0.798
Risanquizumab	0 (0.0%)	12	–	0.0%	
Secuquinumab	4 (80.0%)	476	0.4 (0.2–0.8)	0.9%	
Tocilizumab	1 (20.0%)	313	1.7	0.3%	
Ustequinumab	0 (0.0%)	78	–	0.0%	
**Rituximab**	**4 (4.8%)**	373	**1.0 (0.3–2.5)**	**1.07%**	–
**Abatacept**	**0 (0.0%)**	393	**–**	**0.0%**	–
**Vedolizumab**	**0 (0.0%)**	42	**–**	**0.0%**	–

N: Number of Tuberculosis Cases. TNFi: Tumor Necrosis Factor Inhibitor; The significance level adopted in the study was 5% (p < 0.05).

^a^The p-value was determined using Pearson’s chi-square test for association analyses;

^b^The p-value was determined using Kruskal-Wallis test for median comparisons;

*Comparison between drug classes: p = 0.138 (Fisher’s exact test).

** Comparison of the duration of biological therapy use until TB diagnosis among therapeutic classes: p = 0.466 (Kruskall-Wallis test).

*** Comparison of the duration of biological therapy use until TB diagnosis among TNFi drugs.: p = 0.570 (Kruskall-Wallis test).b.

****The incidence of Tuberculosis with infliximab showed a statistically significant difference when comparing all drugs (p < 0.05), except rituximab (p = 0.112).

### Clinical presentation of tuberculosis

Pulmonary TB was the predominant clinical manifestation, identified in 147 cases (80.8%), while extrapulmonary TB was observed in 28 cases (15.4%). Simultaneous pulmonary and extrapulmonary TB occurred in 7 cases (3.8%). Among the extrapulmonary cases, pleural and bone TB were the most common forms, accounting for 8 cases (22.9%), followed by lymph node TB in 6 cases (17.1%). No significant differences were noted in TB clinical presentation when stratified by timing of diagnosis (pre-therapy or during biological therapy use) or across IMD subgroups (Table 5).

**Table 5:** Clinical characteristics of the 182 cases of Tuberculosis in patients with immune-mediated diseases (IMDs), in Pernambuco, between 2013 and 2023.

Features^a^	General (N = 182)	Rheumatological Diseases (N = 155)	Gastrointestinal Diseases (N = 23)	Dermatological Diseases (N = 4)	P Value^b^
**Forms of Tuberculosis**					0.625
Pulmonary	147 (80.8%)	122 (78.7%)	21 (91.3%)	4 (100%)	
Extrapulmonary	28 (15.4%)	26 (16.8%)	2 (8.7%)	0 (0%)	
*Pleural*	8 (22.9%)	7 (21.2%)	1 (50.0%)	–	
*Peripheral Ganglionar*	6 (17.1%)	6 (18.2%)	0 (0%)	–	
*Bone*	8 (22.9%)	8 (24.2%)	0 (0%)	–	
*Ocular*	1 (2.4%)	1 (2.6%)	0 (0%)	–	
*Miliary*	2 (5.7%)	2 (6.1%)	0 (0%)	–	
*Meningoencephalic*	4 (11.4%)	4 (12.1%)	0 (0%)	–	
*Cutaneous*	1 (2.9%)	0 (0%)	1 (50.0%)	–	
*Other*	6 (17.1%)	6 (18.2%)	0 (0%)	–	
Pulmonary and Extrapulmonary	7 (3.8%)	7 (4.5%)	0 (0%)	0 (0%)	
**Regarding the outcome**					0.113
Complete treatment	131 (59.3%)	108 (57.4%)	17 (81.0%)	2 (50.0%)	
Incomplete treatment (Abandonment)	34 (18.7%)	32 (20.6%)	1 (4.8%)	1 (25.0%)	
Death from Tuberculosis	4 (2.2%)	4 (2.6%)	0 (0%)	0 (0%)	
Death from other causes	11 (6.1%)	11 (7.1%)	0 (0%)	0 (0%)	
Not reported	2 (1.1)	0	2	0	

N: Number of Tuberculosis Cases.

^a^Difference between IMD groups (rheumatological. gastrointestinal. dermatological).

^b^The p-value was determined using Pearson’s chi-square test for association analyses and the Kruskal-Wallis test for median comparisons. The significance level adopted in the study was 5% (p < 0.05).

Of the 182 TB cases recorded among IMD patients, 108 patients (59.3%) completed the treatment, and 34 (18.7%) did not complete the treatment (dropout). TB treatment leaving was documented in 34 patients (18.7%), with the highest rate occurring in the IMRD subgroup (32 cases, 20.6%). TB-related deaths occurred in 4 patients (2.2%), exclusively within the IMRD subgroup.

## Discussion

The occurrence of TB observed in this study among patients with IMD was 2.1%, with 0.9% of TB events occurring during biological therapy use. Interestingly, this is comparable to the TB frequency noted prior to the initiation of biological therapy (1.1%). Several factors could account for this similarity, including the endemic nature of TB in high-incidence regions, the concurrent use of other immunosuppressive medications (such as corticosteroids, azathioprine, and methotrexate), and the immune dysregulation inherent to IMDs. These factors collectively predispose individuals to latent TB reactivation or new TB infections. It is likely that a combination of these mechanisms contributes to the overall risk of TB development among IMD patients [1,2,4,24–26]. However, the absence of detailed pre-biological therapy medication data in our database limits the ability to conduct further risk stratification or comparative analyses concerning prior therapies.

The findings of this study differ from the incidence rates of a nationwide database analysis conducted in South Korea, which focused on patients with Inflammatory Bowel Disease (IBD). The Korean study reported a TB incidence of 0.5% (204/38,830) among IBD patients, and a TB incidence of 2.6% among anti-TNF users [24]. Despite a TB incidence rate of 39 cases per 100,000 inhabitants in South Korea the considerably higher TB incidence in Pernambuco may explain the discrepancy between these findings and our study results.

Among the 83 TB cases identified during biological therapy, 89.2% were associated with the use of anti-TNF agents, most frequently prescribed therapy in the cohort, corresponding to an incidence rate of 1.1% in patients treated with this class of drugs. This incidence is consistent with the results of a meta-analysis study on anti-TNF users, which demonstrated variable TB incidence rates depending on the regional TB burden: 0.02% in countries with low burden (<10 cases per 100,000 population), 0.21% in intermediate-burden regions (10–40 cases per 100,000 population), and 1.59% in high-burden regions (>40 cases per 100,000 population) [4]. These results highlight the strong interplay between environmental TB exposure and the elevated risk of TB in patients undergoing anti-TNF therapy.

In Brazil, Sartori et al. analyzed public drug distribution records for IMRD patients and identified 43 TB cases among 5,853 anti-TNF users over a 10-year period, yielding a incidence of 0.7% [10]. These findings further support higher TB risk in IMD patients on anti-TNF therapy in endemic regions.

In the present study, the risk of TB varied among individual drugs within the anti-TNF class. Infliximab exhibited the highest TB incidence, at 2.6%, a rate significantly greater than other drugs in the same class. This elevated TB risk appears to be related to latent TB infection (LTBI) reactivation, which is of particular concern among patients treated with anti-TNF agents due to the critical role of TNF-α in host resistance against mycobacterial infections.

Among anti-TNF agents, infliximab has been reported to carry higher TB reactivation risk compared to etanercept, which appears to confer relatively lower risk in clinical studies [9,11,13]. The lower TB risk observed with etanercept may be attributed to its mechanism of action: as a fusion protein with two extracellular domains of TNF receptors, etanercept fully inhibits TNF-R2-mediated pro-inflammatory responses but has reduced inhibition of Tumor Necrosis Factor Receptor-1 (TNFR1) signaling. This partial inhibition of TNFR1 may preserve some granuloma functionality, thereby mitigating the risk of TB reactivation compared to monoclonal antibodies like Infliximab or adalimumab [8,10,27,28].

Previous studies have demonstrated varying conclusions regarding the risk of TB across another anti-TNF agents, with some failing to identify significant differences in TB risk among these drugs [9,11–13,15]. For example, a meta-analysis study conducted by Khelghati et al., involving 22,212 patients with rheumatic diseases, found no statistically significant differences in TB risk among various anti-TNF agents, including etanercept and golimumab [14]. Similarly, a South Korean database analysis noted comparable TB incidence rates between etanercept and other anti-TNF therapies, findings that align with the results of the present study. Among 4,736 rheumatoid arthritis patients, infliximab demonstrated an elevated TB risk, with an incidence rate ratio of 2.71 (95% CI: 1.05–7.01). However, the incidence of TB was similar among etanercept, golimumab, and adalimumab [13]. Real-world observational studies further corroborate these findings, reporting no significant differences in TB risk between etanercept and adalimumab, suggesting the mechanism of action of individual drugs may play less of a role in endemic regions [9,11,12].

Another factor to consider is the impact of evolving screening practices. Over the past decade, mandatory TB infection screening prior to initiating biologic therapy has become routine in many countries, with subsequent LTBI treatment substantially altering the risk profile of biologics. Older studies, conducted during periods of less stringent screening protocols, often reported higher TB risks associated with biologics such as Infliximab and Adalimumab when compared to etanercept [8,11,15]. For instance, analysis of Taiwanese data from 1999 to 2009 revealed that Adalimumab carried 2.5 times the risk of TB compared to etanercept. However, subsequent research spanning the period 2000–2015 reported no significant differences in TB incidence across these drugs in the same region, attributing the change to stricter screening guidelines introduced after the initial study [11]. This emphasizes the role of standardized screening protocols in mitigating TB risks across biologic therapies. As newer biologics gain widespread use and more extensive real-world evidence becomes available, future studies may be able to provide a clearer comparative risk profile across drug classes, particularly under contemporary screening and treatment protocols.

In the present study, clinical manifestations of TB during biologic therapy were predominantly pulmonary, accounting for 80.8% of cases, while only 15.4% involved extrapulmonary forms. Among extrapulmonary TB cases, pleural was the most prevalent, followed by bone and lymph node TB. The overall TB-related mortality rate was reported as 2.2%, consistent with findings from studies conducted in referral centers and national registries, which often emphasize frequency without detailed descriptions of clinical presentations, particularly extrapulmonary manifestations [16,24,25,29].

The frequency and severity of extrapulmonary TB manifestations reported in the literature vary widely depending on study design, population characteristics, and healthcare setting [7,12,14,18,30]. For instance, Keane et al. analyzed TB-related adverse events in patients with Crohn’s Disease (CD) treated with anti-TNF therapies soon after their approval by the U.S. Food and Drug Administration (FDA). Among the first 70 reported cases (1998–2001), extrapulmonary TB comprised 57% of cases, with disseminated TB being the most common form (24%; 17/70), followed by lymph node TB (15%; 11/70). This study also highlighted a TB-related mortality rate of 5.7%, emphasizing the severity of extrapulmonary TB in this population [30]. The BiobadaBrasil registry, which tracks biologic therapy outcomes, reported five TB cases (0.5%) among 942 biologic users. Notably, 40% of cases were pulmonary TB, while 60% were extrapulmonary, including one case of disseminated TB and two cases of lymph node TB [12].

On the other hand, studies using medication dispensing databases or case series with a larger number of patients exposed to biological therapy have shown a lower frequency of extrapulmonary forms. Similarly, a South Korean study analyzing 823 IMID patients identified 11 TB cases, with 72% being pulmonary forms, and reported two fatalities [16]. In Brazil, Sartori et al. reported 43 TB cases among anti-TNF users, of which only 30% involved extrapulmonary disease, along with two TB-related deaths (4.6%). However, this study provided limited data on the specific manifestations of extrapulmonary TB [10].

A potential explanation for the higher frequency of extrapulmonary TB observed in referral centers or registries, as opposed to population-based studies, may lie in patient severity. Referral centers often manage patients with more advanced diseases who possess additional risk factors, such as high-dose corticosteroid use, comorbidities, or prolonged immunosuppression. These factors can compromise granuloma integrity, facilitating hematogenous dissemination of *Mycobacterium tuberculosis* and increasing the likelihood of extrapulmonary involvement [7,12,30].

A higher incidence of tuberculosis was observed among patients with axial spondyloarthritis (AS) compared to those with other rheumatologic diseases. However, the literature on infection risk across different rheumatologic conditions remains inconsistent. While some studies suggest a higher risk of TB among patients with rheumatoid arthritis (RA) [14], others report no significant differences between these diseases [6,10,31].

In our analysis of age distribution within the database, we found that patients with AS tended to be younger. This younger demographic may partly explain the higher TB incidence, potentially due to increased environmental exposure in this age group. Supporting this, Souza et al., in an analysis conducted between 2001 and 2020 in the same region as our study, demonstrated that the highest TB incidence occurred among younger individuals, with a predominance in those aged 20–39 years (43%), followed by those aged 40–59 years (32%). These findings align with the age distribution observed among patients with AS in our study. In contrast, patients with RA predominantly belonged to the 60 years or older age group [32].

This finding was consistent when comparing demographic characteristics between TB and non-TB groups, with the TB group showing a lower median age and a higher frequency in age groups up to 49 years, reinforcing that younger individuals are at higher risk.

This study revealed a higher incidence of TB among patients with IMGD compared to psoriasis patients, with the lower rates observed in psoriasis likely attributable to differences in treatment protocols and the degree of immunosuppression. One hypothesis for the higher frequency found is that in conditions such as Inflammatory Bowel Disease (IBD), high levels of immunosuppression are commonly induced by the use of drugs like azathioprine, methotrexate, and corticosteroids (>40 mg/day), especially during disease flares. Furthermore, the combination of Infliximab with azathioprine, a common therapeutic approach in IBD management, significantly increases immunosuppression and, consequently, the risk of opportunistic infections, including TB [25,26,33–35]. Marehbian et al. conducted a study investigating treatment-related adverse events in 22,310 Crohn’s Disease patients and 111,550 controls, comparing risks associated with corticosteroids, immunosuppressive drugs, and biologic monotherapy. The analysis revealed that the risk of TB with monotherapy had a risk ratio (RR) of 2.7 (95% CI: 1.0–7.3), which was considerably lower than the TB risk associated with combination therapy (RR: 27.4; 95% CI: 2.1–26.3) [26]. A cohort study by Brito et al. conducted in Northeastern Brazil, involving 571 IMIG disease, revealed that 60% of participants were receiving immunosuppressive drugs, with 47% of biologic users on concomitant treatment with azathioprine or methotrexate [33]. These practices may exacerbate immunosuppressive effects, contributing to the higher incidence of TB observed in IMIDs.

Moreover, the greater impairment of nutritional status due to intestinal malabsorption and blood loss in patients with IBD may also contribute to immune deficiency and favor a higher occurrence of TB. However, the lack of detailed data on concomitant medications or nutritional status in our database does not allow us to verify these hypotheses, highlighting the need for future studies based on medical records from referral centers with more comprehensive clinical data.

In contrast, psoriasis treatment protocols, particularly pre-biologic therapy, often emphasize non-immunosuppressive or corticosteroid-free therapies, which likely explains the lower TB prevalence seen among these patients. Dermatology tends to rely on treatments with mechanisms that pose minimal risk for latent TB reactivation or opportunistic infections. This distinction underscores the critical role of both drug-specific mechanisms and prescribing practices in modulating TB risk across IMID subtypes. In rheumatology, despite substantial use of immunosuppressive drugs, treatment often incorporates non-immunosuppressive disease-modifying therapies, alongside lower doses of corticosteroids than those typically used in IBD management. These treatment strategies likely mitigate the level of immunosuppression in rheumatologic patients compared to those with IBD, contributing to the observed differences in TB incidence between these groups [36,37].

One potential factor contributing to the lower frequency of TB in psoriasis patients is the reduced rate of false positives associated with the Tuberculin Skin Test (TST), likely related to lower levels of immunosuppression. This improves the accuracy of Latent TB Infection (LTBI) detection, enabling more effective prophylaxis and reducing the risk of reactivation after biologic initiation. Studies consistently report higher TST positivity rates in psoriasis compared to other IMIDs [38,39]. This may be explained by enhanced Th1/Th17 activation and the presence of tissue-resident memory T cells (TRM) pathways observed in psoriasis, which strengthen immune responses against LTBI [40–42]. Additionally, the lower use of high-dose corticosteroids and the limited impact of psoriasis-specific methotrexate dosing on immune reactivity help preserve TST accuracy [43–45]. Together, these factors may improve LTBI detection and management, contributing to a lower TB risk in this population.

This study has several limitations. The database contains standardized epidemiological and clinical notification forms but does not provide information on relevant covariates such as socioeconomic status, body mass index, nutritional status, comorbidities, or the concomitant use of other immunosuppressive agents among patients receiving biologic therapy, all of which may influence TB risk across different IMD. Additional multicenter studies based on patient-level clinical data across different healthcare institutions in the region may help to better evaluate these factors and further explore the observed differences.

One of the strengths of our study is the large number of patients receiving biologic therapy included in the analysis, using a centralized public drug distribution system that covers approximately 85% of the population in the region over a 10-year period. Given that tuberculosis is a notifiable disease in Brazil and that its treatment is provided exclusively through the public health system, the recorded data used to estimate incidence and describe clinical patterns are considered reliable. Despite potential limitations inherent to the database, the available data provide valuable insights into TB incidence across different IMD receiving biologic therapy in endemic region, as well as the clinical patterns of infection, helping to address existing regional knowledge gaps. The comparison of three IMD groups, which is still scarcely described in the literature, allowed us to highlight relevant differences and emphasize the need for further studies.

In summary, this study observed a similar frequency of tuberculosis (TB) before and during biologic therapy, likely influenced by high regional TB endemicity, other covariates not evaluated in the study may also be involved, such as concurrent immunosuppressive use, and immune dysregulation inherent to immune-mediated inflammatory diseases (IMIDs). The highest proportion of TB was identified in patients with gastroenterological diseases. Pulmonary TB was the most prevalent form among biologic users, while extrapulmonary TB cases were generally less severe. No significant differences in TB incidence were found among anti-TNF agents and other cytokine inhibitors, except for infliximab. These findings underscore the importance of large-scale, multicenter studies to investigate TB patterns across IMID populations and regions, supporting optimized screening, prophylaxis, and tailored guidelines for biologic therapy in TB-endemic areas.

## Supporting information

S1 Table Comparison of tuberculosis incidence among subgroups of in patients with Immune Mediated Diseases (IMDs) based on demographic variables available in the dataset, such as age. (PDF)

S2 Table Tuberculosis incidence before and after treatment in the Tumor Necrosis Factor Inhibitor (TNFi) medication class group. (PDF)
