# Supplementary material for: Incidence of tuberculosis in patients with immune-mediated diseases undergone biological therapy: A 10-year observational study in a high-burden region of northeastern Brazil
Source: PLoS One. 2026 Jul 27;21(7):e0353691. doi: 10.1371/journal.pone.0353691 (PMC13405316; doi:10.1371/journal.pone.0353691)
Supplement: S1 Table — (PDF) [file pone.0353691.s001.pdf]

S1 Table. Comparison of tuberculosis incidence among subgroups of in patients with Immune-Mediated Diseases (IMDs) based on demographic variables available in the dataset, such as age.

Rheumatological Diseases	Rheumatoid arthritis	Psoriatic arthritis	Axial spondyloarthritis	P Value[a]
Age				
Median (P_{25} – P_{75})	57 (47 – 65)	56 (46 – 64)	**45 (36 – 54)**	<0,001[a]
Age Range				<0,001
Up to 39 y/o	573 (12,0%)	111 (13,0%)	**737 (33,8%)**	
40 to 49 y/o	870 (18,2%)	160 (18,8%)	622 (28,5%)	
50 to 59 y/o	1,266 (26,4%)	244 (28,6%)	531 (24,4%)	
60 years y/o	**2,080 (43,4%)**	**337 (39,6%)**	291 (13,3%)	

[a]Statistically significant difference between rheumatoid arthritis and psoriatic arthritis when compared to axial spondyloarthritis ($p < 0.001$).
