# Supplementary material for: Incidence of tuberculosis in patients with immune-mediated diseases undergone biological therapy: A 10-year observational study in a high-burden region of northeastern Brazil
Source: PLoS One. 2026 Jul 27;21(7):e0353691. doi: 10.1371/journal.pone.0353691 (PMC13405316; doi:10.1371/journal.pone.0353691)
Supplement: S2 Table — (PDF) [file pone.0353691.s002.pdf]

S2 Table. Tuberculosis incidence before and after treatment in the Tumor Necrosis Factor Inhibitor (TNFi) medication class group.

**Groups**[a]	**Before Treatment**[b] N/Total (% - CI 95%)	**During Treatment**[c] N/Total (% - CI 95%)	**P Value**[d]
**TNFi**	65/6.862 (0.95 – 0.74 a 1.21)	74/6.871 (1.08 – 0.85 a 1.35)	0.447
Adalimumab	21/2.850 (0.74 – 0.48 a 1.12)	26/2.855 (0.91 – 0.62 a 1.33)	0.478
Certolizumab	11/632 (1.74 – 0.96 a 3.11)	6/627 (0.96 – 0.43 a 2.11)	0.231
Etanercept	9/1,608 (0.56 – 0.29 a 1.07)	13/1.621 (0.81 – 0.47 a 13.8)	0.389
Golimumab	12/1,003 (1.20 – 0.68 a 2.09)	9/1.000 (0.90 – 0.47 a 1.72)	0.510
Infliximab	12/769 (1.56 – 0.88 a 2.73)	20/777 (2.57 – 1.66 a 3.95)	0.163

N: Number of Tuberculosis Cases.

TNFi: Tumor Necrosis Factor Inhibitor

[a]Group of patients receiving TNFi and found that the risk of TB did not differ before versus during biologic therapy, as shown below.

[b]Incidences by TNFi medication class group (Adalimumab, Certolizumab, Etanercept, Golimumab and Infliximabe) before treatment.

[c]Incidences by TNFi medication class group (Adalimumab, Certolizumab, Etanercept, Golimumab and Infliximabe) during treatment.

[d]The p-value was determined using Pearson's chi-square test for association analyses and the Kruskal-Wallis test for median comparisons. The significance level adopted in the study was 5% ($p<0.05$).
